# Supplementary material for: Molecular basis for recognition and deubiquitination of 40S ribosomes by Otu2
Source: Nat Commun. 2023 May 12;14:2730. doi: 10.1038/s41467-023-38161-w (PMC10175282; doi:10.1038/s41467-023-38161-w)
Supplement: Supplementary file 1 — Supplementary Information [file 41467_2023_38161_MOESM1_ESM.pdf]

Molecular basis for recognition and deubiquitination of 40S ribosomes by Otu2

Authors: Ken Ikeuchi, Nives Ivic, Robert Buschauer, Jingdong Cheng, Thomas Fröhlich, Yoshitaka Matsuo, Otto Berninghausen, Toshifumi Inada, Thomas Becker and Roland Beckmann

Supplementary Information

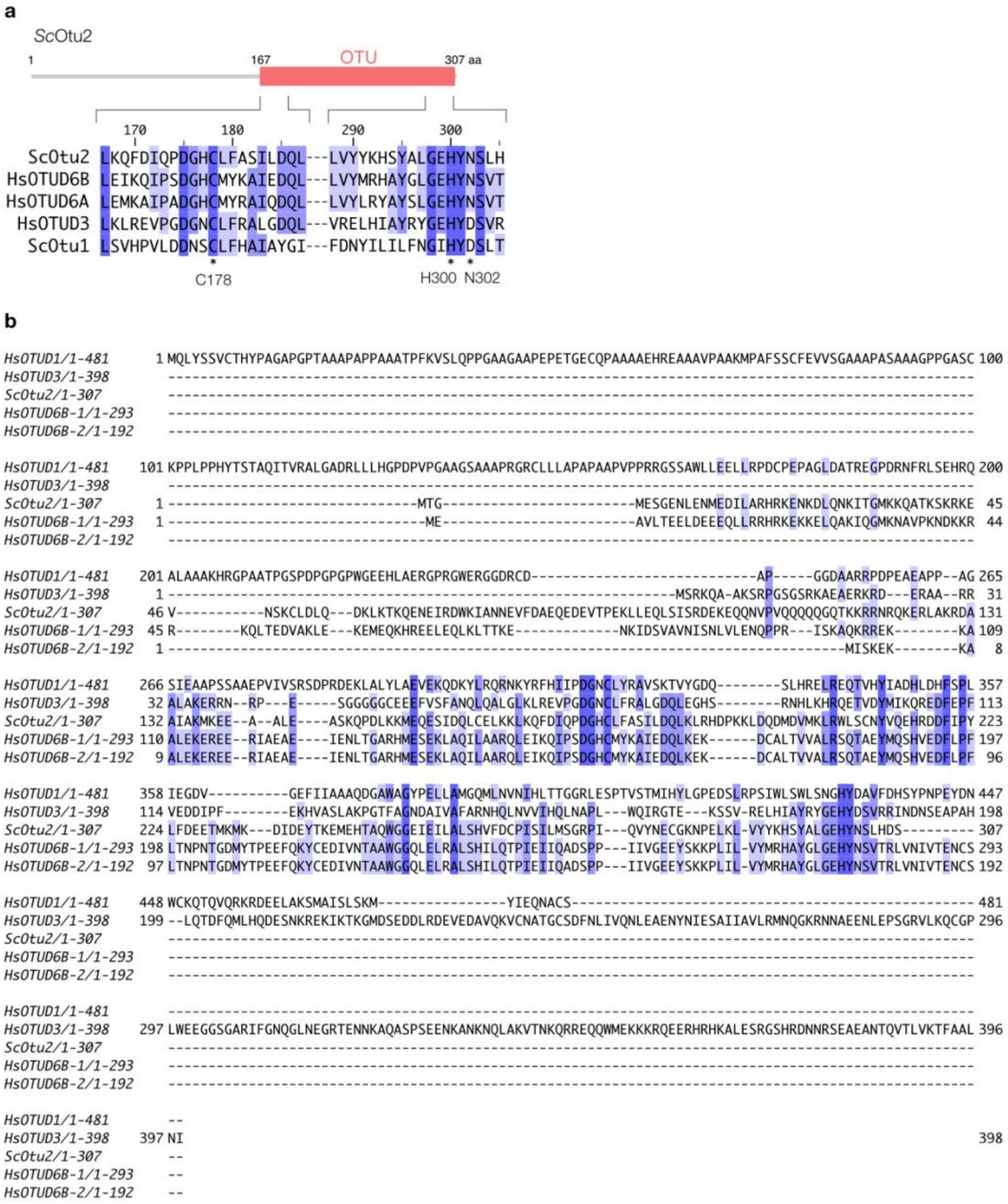

Supplementary Fig. 1. Sequence alignment of OTU domain containing proteins.

a, Sequence alignment of the regions flanking the catalytic triad residues. **b**, Complete sequence comparison of OTU domain containing proteins. Like *H. s.* OTUD1 and OTUD3, *S.c.* Otu2 has an N-terminal S1’α-helix (Leu150-Lys165).

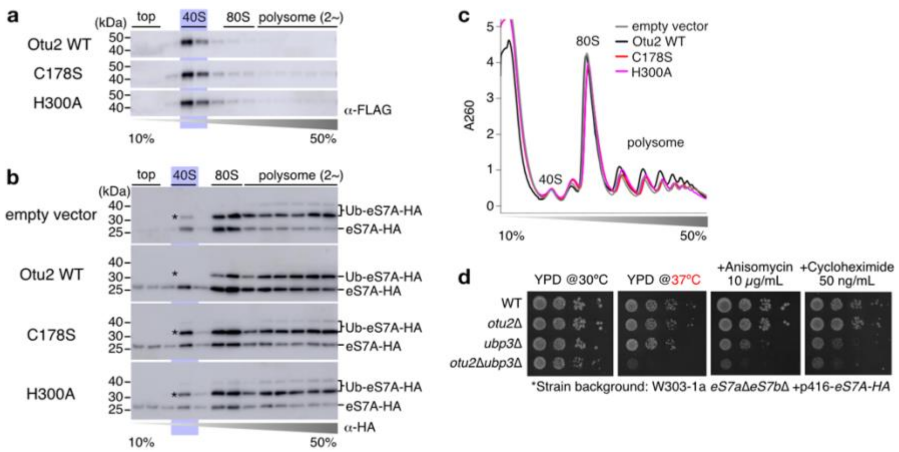

Supplementary Fig. 2. 40S association of Otu2 and its catalytic mutants.

a, Western blot analysis of fractions after sucrose density gradient centrifugation. Lysates were analyzed from an *otu2*Δ with shuffled *eS7A-HA* yeast strain harboring a vector expressing wild type (wt) Otu2-3xFLAG, otu2-C178S-3xFLAG and otu2-H300A-3xFLAG, respectively. Western blots were done using α-FLAG. We obtained essentially the same results in at least three independent experiments. **b**, Western blot analysis of fractions after sucrose density gradient centrifugation. Lysates were analyzed from an *otu2*Δ*ubp3*Δ with shuffled *eS7A-HA* yeast strain harboring an empty vector, a vector expressing wild type (WT) Otu2, otu2-C178S and otu2-H300A, respectively. Western blots were done using α-HA. Asterisks indicate mono-ubiquitinated eS7 in the 40S fraction. We obtained essentially the same results in at least three independent experiments. HA; hemagglutinin. **c**, UV profiles corresponding to **b** after sucrose density gradient centrifugation. **d**, Spot assay of yeast mutant cells in a series of 10x dilution grown for 2 days under the stress conditions. Source data for (**a**) and (**b**) are provided in the Source data file. YPD; Yeast extract Peptone Dextrose medium.

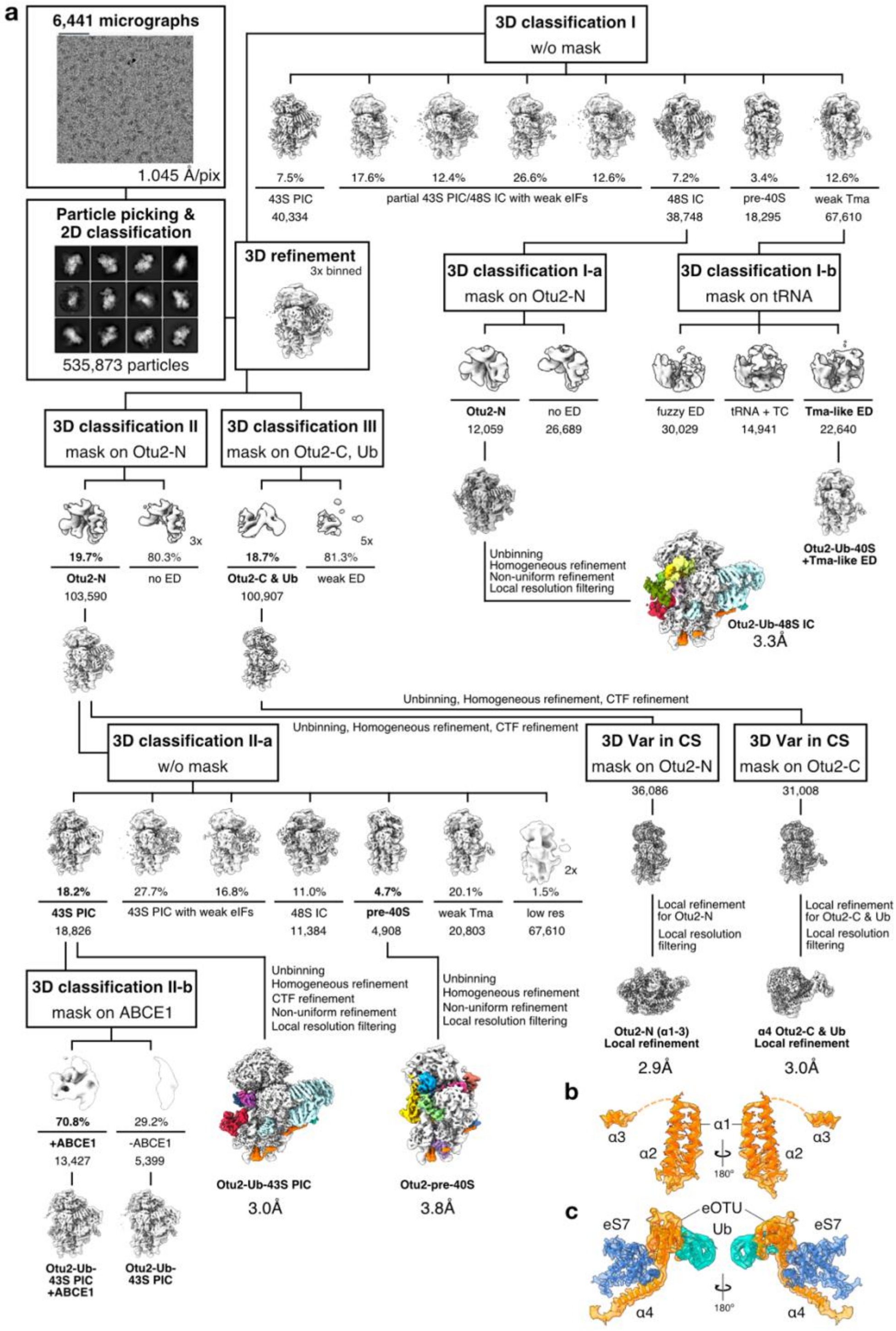

Supplementary Fig. 3. Data processing scheme for cryo-EM analysis of native Otu2-40S complexes.

a, From a total of 6,441 micrographs, after 2D classification, 535,873 particles were refined and subjected to two branches of 3D classification, once without a mask (Classification I) and once with soft masks around the binding region of the Otu2 N-terminal (Classification II) and C-terminal (Classification III) domains (Otu2-N and Otu2-C; see also Fig. 2e) in RELION-3.1[1]. Classification I revealed 40S ribosomal particles including pre-40S, 43S and partial 48S particles. One class (denoted as "weak Tma") contained extra density in a position previously shown to be occupied by human homologs of mRNA recycling factors Tma20 (MCT-1), Tma22 (DENR) and Tma64 (eIF2D). Further subclassification after classification I yielded in a stable 48S-IC containing Otu2 bound to eS7-Ub and

ABCE1 as well as one class with enriched extra density presumably for Tma proteins (“Tma-like ED”). Subclassification after classifications II and III yielded in a stable 43S-PIC that was further sub-classified for ABCE1 presence and in a homogenous pre-40S class. All stable classes were refined and to high resolution and post-processed in CryoSPARC v3.3.2.
Further, all particles with extra density for Otu2-N and Otu2-C, respectively were subjected to 3D variability analysis and focused refinement in CryoSPARC, resulting in best-resolved structures of 40S-bound Otu2-N (α1-α3; **b**) and the OTU-domain bound to Lys83-ubiquitinated eS7 and ubiquitin (**c**). See Materials and Methods for details.

b, molecular model for α1-α3 of Otu2-N fit into the focused refined density.

c, molecular model for eS7, α4 and eOTU of Otu2 and ubiquitin fit into the focused refined density.

ED, extra density; Ub, ubiquitin; CS, CryoSPARC; PIC, pre-initiation complex; IC, initiation complex; eOTU, extended OTU domain.

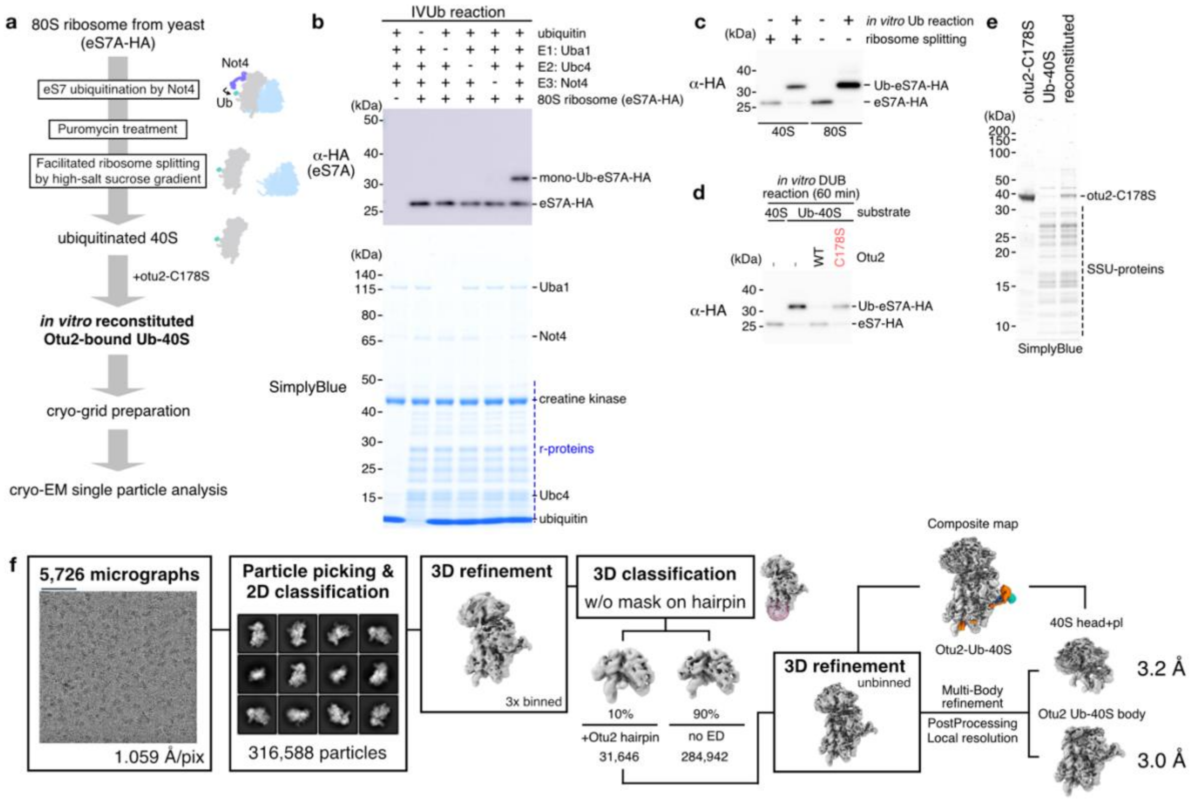

Supplementary Fig. 4. In vitro monoubiquitination of eS7 in 80S ribosomes

a, Scheme outlining the in vitro generation of eS7A-monoubiquitinated 40S and cryo-EM sample preparation. **b**, α-HA Western blot monitoring in vitro generation of eS7A-monoubiquitinated 80S ribosomes. Below, a SimplyBlue-stained Nu-PAGE gel of the input reactions is shown. r-proteins; ribosomal proteins. HA; hemagglutinin. **c**, α-HA Western blot showing eS7A-ubiquitinated 40S after puromycin/high salt induced splitting of 80S ribosomes. **d**, α-HA Western blot showing in vitro deubiquitination reactions with eS7A-monoubiquitinated 40S and wt Otu2 or otu2-C178S mutant proteins. **e**, Nu-PAGE gel showing the Otu2-Ub-40S cryo-EM sample in vitro reconstituted from purified eS7A-monoubiquitinated 40S subunits and otu2-C178S mutant protein. For all experiments displayed in (**b**) to (**e**) we obtained essentially the same results in at least three independent experiments. SSU; small subunit. Source data for (**b**), (**c**), (**d**) and (**e)** are provided in the Source data file.

f, Cryo-EM data processing scheme for in vitro reconstituted Otu2-Ub-40S complex. The dataset was processed with RELION-3.1[1]. From a total of 5,726 micrographs, after 2D classification, 316,588 particles were selected and refined. Focused classifications using a soft spherical mask were performed on 40S foot region showing extra density (ED) for the Otu2 N-terminal hairpin. Classes with enriched Otu2 density were refined followed by multi-body refinement for the 40S head and 40S body region (including Otu2). This resulted in final reconstructions at an average resolution of 3.0Å for the Otu2-containing 40S body and 3.2Å for the 40S head. See Materials and Methods for details.

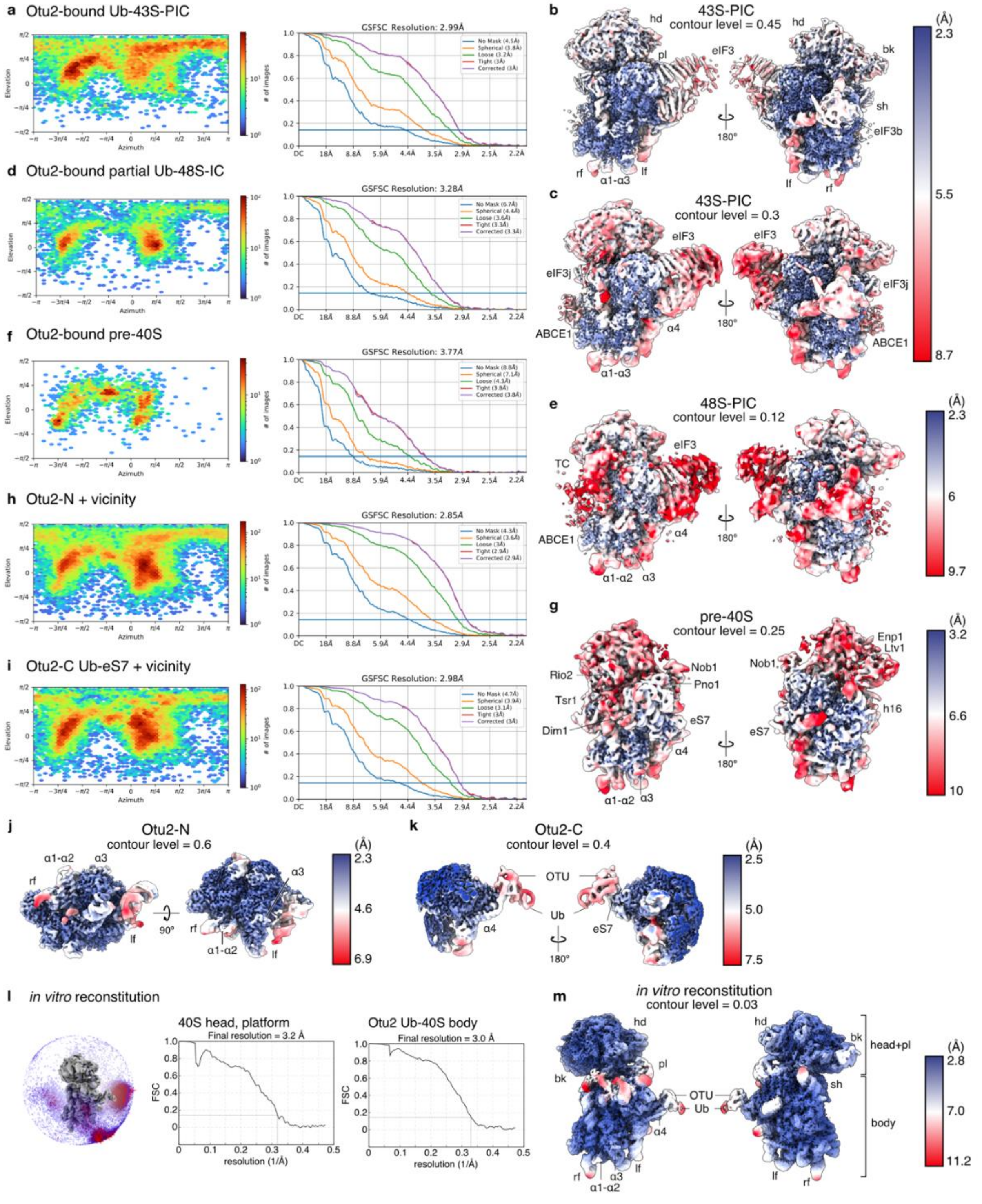

Supplementary Fig. 5. Resolution of Otu2-40S cryo-EM maps.

Shown are angular distribution plots, Fourier shell correlation (FSC) curves and electron potential maps of refined Otu2-bound 40S ribosomal particle classes (see also Supplementary Figs. 3 and 4f). **a-c**, Angular distribution plot and FSC curve (**a**) and maps shown at the indicated contour level (**b**, **c**) for the Otu2-Ub-43S-PIC. **d-e**, Angular distribution plot and FSC curve (**d**) and map (**e**) for the Otu2-Ub-48S-IC. **f-g,** Angular distribution plot and FSC curve (**f**) and map (**g**) for the Otu2-pre-40S. **h-k,** Angular distribution plots and FSC curves after local refinement for Otu2-N (**h**) and Otu2-C (**i**) binding region and corresponding masked cryo-EM maps shown in two orientations (**j,** Otu2-N; **k,** Otu2-C). **l**, Angular distribution plot and FSC curves after multi-body refinement of the in vitro reconstituted Otu2-Ub-40S

complex in RELION-3.1. **m**) and composite map of multi-body refined 40S head and 40S body shown in two orientations.

All maps were refined, low-pass filtered and colored according to local resolution in CryoSPARC v3.3.2 and ChimeraX (v.1.3) except for the in vitro reconstituted Otu2-40S complex (processed with RELION-3.1). All angular distribution plots and were exported from CryoSPARC v3.3.2 except for **i**), which was exported from RELION-3.1. The average resolution was calculated according to the gold standard criterion at FSC = 0.143 (GSFSC) and plots were exported from CryoSPARC v3.3.2, except for **i**), which was derived from RELION-3.1.

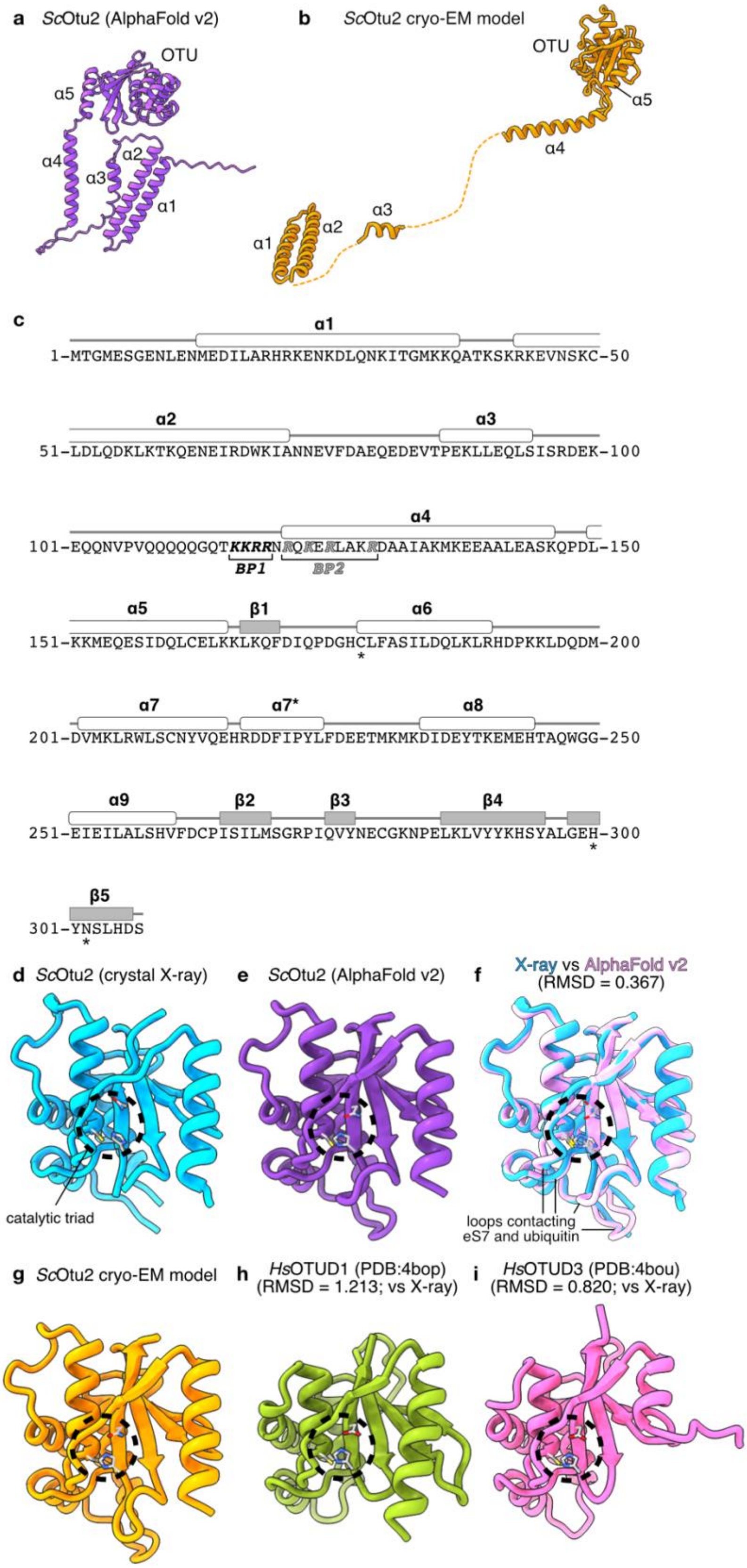

Supplementary Fig. 6. Prediction of secondary structure and structural comparison for Otu2.
a, A model of full length Otu2 predicted by AlphaFold v2.0 (AF-2)[2]. α1, α2, α4 and extended OTU domain showed higher per-residue confidence score (pLDDT > 70), while α3 showed low confidence (predicted local-distance difference test (pLDDT) < 70). **b**, Structural model of 40S-bound Otu2 as

determined by cryo-EM. **c**, Schematic summary of secondary structure prediction by AF2. α-helices are labeled according to their occurrence in the cryo-EM structure. Residues of the catalytic triad are indicated by asterisks. **d-i**, Structural models of the OTU domain of Otu2 and related OTU domain-containing deubiquitinases. **d**, *S. c.* Otu2 crystal structure from this study. **e**, *S. c.* Otu2 structure predicted by AF2. **f**, Comparison between the *S. c.* Otu2 crystal structure and AF2 model. While being highly similar, differences occur in the indicated loop regions that contact eS7. **g**, *S. c.* Otu2 cryo-EM model from this study. **h**, *H. s.* OTUD1 (PDB:4bop). **i**, *H. s.* OTUD3 (PDB:4bou) [3]. RMSD values in **f**, **h** and **i** refer to a comparison with the Otu2 crystal structure.

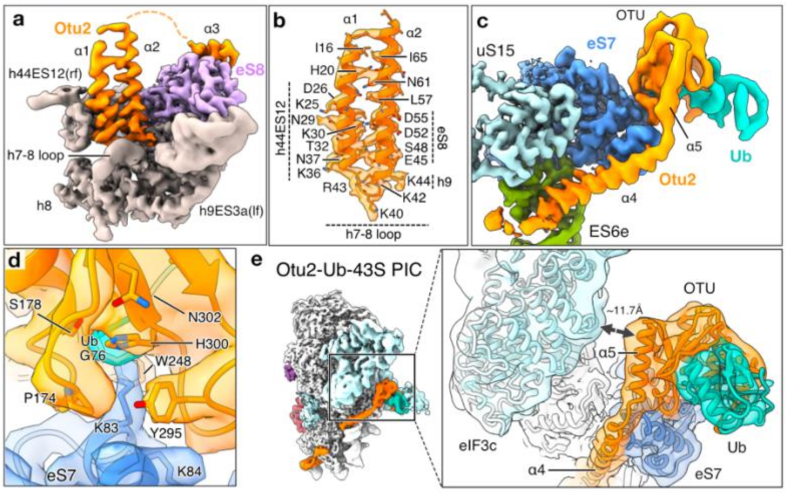

Supplementary Fig. 7. High resolution structure of Otu2 bound to the 40S subunit and position within the 43S-PIC.

a, Electron potential map after focused classification and refinement on the Otu2-N binding region (see Supplemental Fig. 3). The view focuses on the terminal hairpin (α1- α2) and α3 to h44 (ES12) and their contact sites to the loop between h7 and h8, h9 (ES3a) and eS8. The view corresponds to Fig. 3a. **b**, Molecular model of the Otu2 α1- α2 hairpin with fitted of the isolated map model. Interacting sites on the 40S are indicated. **c**, Cryo-EM map after focused classification and refinement on the Otu2-C (see Supplemental Fig. 3). The view focuses on the extended Otu2 OTU domain and α4 bound to Lys83-monoubiquitinated eS7 and ES6e. The view corresponds to Fig. 3d. Ub; ubiquitin. **d**, Cryo-EM map and fitted model for the eS7-Lys83 ubiquitination site shown as in Fig. 3f. **e**, Cryo-EM map of the Otu2-Ub-43S-PIC and zoom view showing the molecular model fitted into the map and focusing on the location of the Ub-bound OTU domain in vicinity to the eIF3c PCI domain.

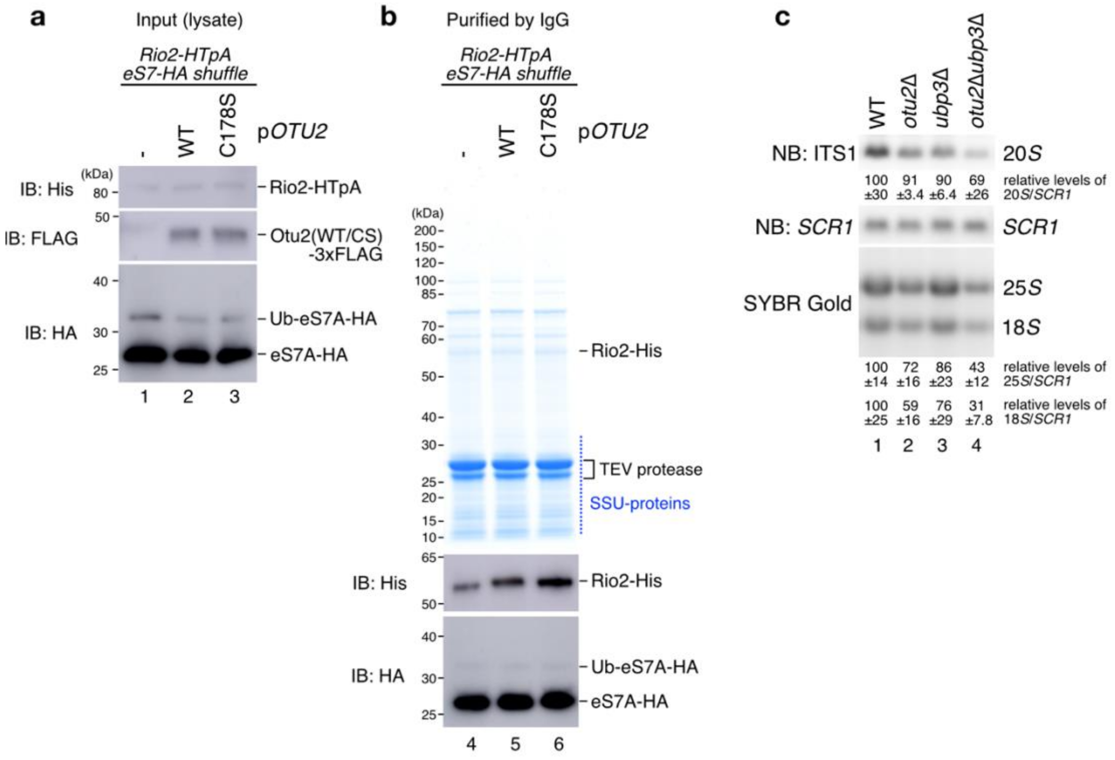

Supplementary Fig. 8. Biochemical characterization of Otu2-bound pre-40S.

a, Western blot analysis of lysates from yeast shuffle strains harboring genomically HTpA-tagged Rio2, an expression plasmid for HA-tagged eS7 and either a vector with wild type (WT) Otu2 (lane 2) or with the catalytic dead Otu2 mutant (C178S; CS, lane 3). Empty vector (lane 1) served as a control. HA; c. **b,** Nu-PAGE gel and Western blot analysis of Rio2 affinity purification from yeast shuffle strains described in **a**. Shown are the elutions with TEV protease. SSU; small subunit. HA; hemagglutinin **c,** Northern blot analysis and SYBR Gold stain of total RNA extracted from eS7-shuffled wild type (WT), *otu2*Δ, *ubp3*Δ or *otu2*Δ*ubp3*Δ yeast cells. As probes, DIG-labeled oligonucleotides against 20S pre-rRNA as well as *SCR1* (as loading control) were used and relative levels compared to the loading controls were calculated. We cannot provide a molecular weight marker for Supplementary Fig. 8c, since a DIG -labeled RNA marker is not commercially available anymore. Other pre-stained markers are only available for small RNAs (<100 bases) to be used for TBE-urea PAGE gels, but not for agarose gels that were used here.

For all experiments displayed in (**a**), (**b**) and (**c**) we obtained essentially the same results in at least three independent experiments Source data for (**a**), (**b**) and (**c**) are provided in the Source data file.

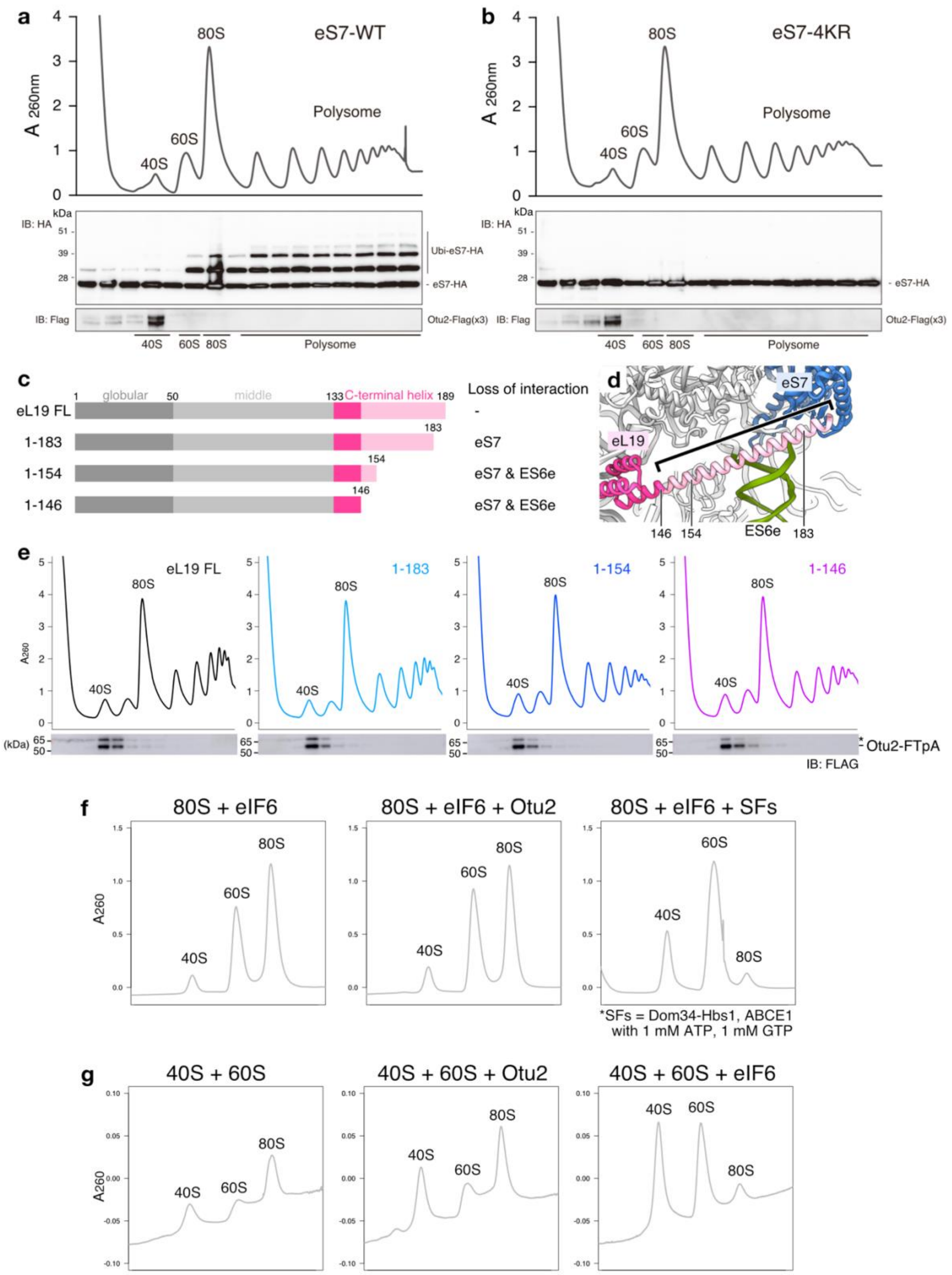

Supplementary Fig. 9. Biochemical characterization of Otu2 association with 40S.

a, b, Western blot analysis after sucrose density gradient centrifugation of cell lysates from yeast strains expressing FLAG-tagged Otu2 and either HA-tagged wild type (WT) eS7 (**a**) or HA-tagged eS7 where all four lysine residues (Lys72, Lys76, Lys83 and Lys84) close to Not4-specific ubiquitination site were mutated to arginine residues (eS7-4KR; **b**)[4]. For all experiments displayed in (**a**) and (**b**) we obtained essentially the same results in at least three independent experiments. HA = haemagglutinine. **c**, Scheme outlining domains and C-terminal deletion constructs for LSU protein eL19. It consists of an N-terminal

globular domain, a middle domain and a C-terminal α-helix that forms intersubunit bridge eB12 with eS7 and rRNA expansion segment ES6e. **d**, molecular model of the eB12 region in the 80S ribosome (PDB: 6snt). eL19 is color coded as in **c**. C-terminal residues of eL19 deletion mutants are indicated in the structural model. **e,** Western blot analysis after sucrose density gradient centrifugation of cell lysates from yeast strains expressing the indicated C-terminal truncation mutants of eL19 mutants and FTpA-tagged Otu2. FTpA = FLAG-TEV-proteinA. The proteins labeled by asterisk seem to be Otu2-FTpA with unidentified modification. We obtained essentially the same results in at least three independent experiments. **f,** Polyribosome profiles of in vitro splitting assays with purified Otu2 (middle) and control profiles without Otu2 (left) or and including *bona fide* splitting factors (SF; Dom34, Hbs1 and ABCE1; right) are shown. **g**, Polyribosome profiles of in vitro re-association assays with purified 40S and 60S subunits. Otu2 (middle) and control profiles without Otu2 (left) or with *bona fide* anti-association factor eIF6 are shown. For all experiments displayed in (**e**), (**f**). and (**g**) we obtained essentially the same results in at least three independent experiments. Source data for (**a**) and (**b**) are provided in the Source data file.

Supplementary Table 1. Data collection, refinement and model statistics.

Supplementary Table 1. cryo-EM statistics

	native otu2-C178S bound complexes					*in vitro* reconstitution
**EMD-IDs**	**EMD-16525**	**EMD-16533**	**EMD-16541**	**EMD-16542**	**EMD-16548**	**EMD-16470, EMD-16471**
**Data collection and Processing**	43S PIC	48S IC	pre-40S	Otu2-N + vicinity	Otu2-C, Ub + vicinity	otu2-C178S-Ub-40S
Magnification	130,000	130,000	130,000	130,000	130,000	130,000
Voltage (kV)	300	300	300	300	300	300
Electron exposure ($e^-/Å^2$)	46.4	46.4	46.4	46.4	46.4	46
Defocus range (μm)	0.5 to 3.0	0.5 to 3.0	0.5 to 3.0	0.5 to 3.0	0.5 to 3.0	0.5 to 4.0
Pixel size (Å)	1.045	1.045	1.045	1.045	1.045	1.059
Symmetry imposed	C_1	C_1	C_1	C_1	C_1	C_1
Micrographs Used (no.)	6,441	6,441	6,441	6,441	6,441	5,726
Initial Particle Images (no.)	656,489	656,489	656,489	656,489	656,489	540,175
Particle Images Used (no.)	535,873	535,873	535,873	535,873	535,873	316,588
Final Particle Images (no.)	18,826	12,059	4,908	36,086	31,008	31,646
Map resolution (Å)	3.0	3.3	3.8	2.9	3.0	3.0 (body) / 3.2 (head)
FSC threshold	0.143	0.143	0.143	0.143	0.143	0.143
Map sharpening *b*-factor ($Å^2$)	-46	-47	-40	-64.1	-66	20
**Refinement and Validation**	**PDB 8CAH**	**PDB 8CAS**	**PDB 8CBJ**	**-**	**-**	**PDB 8C83**
Initial Models Used	6ZCE (43S PIC)	6ZU9 (48S PIC), 6FYY (eIF2)	6FAI (pre-40S), 6RBD (Dim1)			6TB3 (40S), 3C0R (Ub), Xtal and AF2 (Otu2)
Model Resolution (Å)	3.0	3.3	3.8			3.1
FSC threshold	0.5	0.5	0.5			0.5
Model Composition						
Nonhydrogen Atoms	95,564	86,738	87,884			45,803
Protein Residues	8,423	8,972	6,469			2,725
Nucleotides	1,745	1,846	1,767			1,177
Ligands	85	14	48			0
R.m.s. deviations						
Bond Length (Å)	0.007	0.006	0.006			0.005
Bond Angles (º)	1.026	0,952	1.154			0.977
Map vs. model CC (mask)	0.78	0.77	0.77			0.83
MolProbity Score	1.57	1.44	1.62			1.66
Clashscore	4.25	2.5	5.45			4.85
Poor Rotamers (%)	0.69	0.93	0.02			0.58
Ramachandran plot						
Favored (%)	94.76	93.89	95.39			93.89
Allowed (%)	5.14	5.99	4.55			6.11
Disallowed (%)	0.11	0.12	0.06			0.00

Supplementary Table 2. Data collection and refinement statistics for the extended OTU domain crystal structure.

Supplementary Table 2. Statistics for X-ray crystal structure

**ScOtu2 extended-OTU domain C178S**	**Otu2 (150-307) C178S PDB 7PL7**
**Data collection**	
Wavelength (Å)	0.979
Space Group	I 21 21 21
Cell dimension	
a, b, c (Å)	44.67, 60.05, 148.35
α, β, γ (º)	90, 90, 90
Resolution (Å)	42.77-2.60 (2.75-2.60)
Total Reflections	86 288
Unique Reflections	11 976
R_{merge}	0.139 (1.038)
$I/\sigma I$	15.1 (3.1)
Completeness (%)	99.7 (98.3)
Redundancy	13.0 (13.6)
CC1/2	0.999 (0.937)
**Refinement**	
R_{work}/R_{free}	0.2252/0.2643
No. Atoms	
Protein	1295
Ligands/Ions	0
Water	27
B-factors	
Protein	74.817
Ligands/Ions	0
Water	67.581
R.m.s. deviations	
Bond Length (Å)	0.003
Bond Angles (º)	0.52
**Validation**	
Ramachandran plot	
Favored (%)	93.29
Allowed (%)	6.04
Disallowed (%) or Outliers (&)	0.67

Supplementary Table 3. Yeast strains used in this study.

Supplementary Table 3. Yeast Strains used in this study.

Strain ID	Name	Genotype	Reference
parental strain	*W303-1a wt*	*MAT* a *ade2 his3 leu2 trp1 ura3 can1*	Lab stock
YKI2086	*OTU2-FTpA*	*OTU2-FTpA-natNT2*	this study
YKI2157	*UBP3-FTpA*	*UBP3-FTpA-natNT2*	this study
YKI2105	*otu2* Δ	*otu2* Δ::*kanMX6*	this study
YKI2161	*leu1* Δ	*leu1* Δ::*kanMX4*	this study
YKI2185	*OTU2-FTpA eL19* -shuffled	*OTU2-FTpA-natNT2, rpl19a* Δ::*kanMX4* , *rpl19b* Δ::*hphNT1* , p416*GPDp-rpl19-CYC1t*	this study
YKI2212	*OTU2-FTpA eL19-full length*	*OTU2-FTpA-natNT2, rpl19a* Δ::*kanMX4* , *rpl19b* Δ::*hphNT1* , p414-*rpl19-CYC1t*	this study
YKI2213	*OTU2-FTpA eL19-1-183aa*	*OTU2-FTpA-natNT2, rpl19a* Δ::*kanMX4* , *rpl19b* Δ::*hphNT1* , p414-*rpl19(1-183)-CYC1t*	this study
YKI2214	*OTU2-FTpA eL19-1-154aa*	*OTU2-FTpA-natNT2, rpl19a* Δ::*kanMX4* , *rpl19b* Δ::*hphNT1* , p414-*rpl19(1-154)-CYC1t*	this study
YKI2215	*OTU2-FTpA eL19-1-146aa*	*OTU2-FTpA-natNT2, rpl19a* Δ::*kanMX4* , *rpl19b* Δ::*hphNT1* , p414-*rpl19(1-146)-CYC1t*	this study
YKI2195	*eS7A-HA* -shuffled *RIO2-HTpA*	*rps7a* Δ::*kanMX6* , *rps7b* Δ::*hphNT1* , p4*16-RPS7A-HA-CYC1t* , *RIO2-HTpA-HIS3MX6*	this study
Y124	*eS7A-HA* -shuffled	*rps7a* Δ::*HIS3MX6* , *rps7b* Δ::*natNT2* , pRS316-*RPS7A-HA-CYC1t*	Ikeuchi *et al* . 2019
Y124 *otu2* Δ	*eS7A-HA* -shuffled *otu2* Δ	Y124, *otu2* Δ::*hphMX4*	this study
Y124 *ubp3* Δ	*eS7A-HA* -shuffled *ubp3* Δ	Y124, *ubp3* Δ::*kanMX6*	this study
Y124 *otu2* Δ*ubp3* Δ	*eS7A-HA* -shuffled *otu2* Δ*ubp3* Δ	Y124, *ubp3* Δ::*kanMX6* , *otu2* Δ::*hphMX4*	this study
eS7A-WT	*eS7A-WT*	*rps7a* Δ::*HIS3MX6* , *rps7b* Δ::*natNT2* , pRS315-*RPS7A-HA-CYC1t*	Ikeuchi *et al* . 2019
eS7A-4KR	*eS7A-4KR*	*rps7a* Δ::*HIS3MX6* , *rps7b* Δ::*natNT2* , pRS315-*rps7A-4KR(K72,76,83,84R)-HA-CYC1t*	Ikeuchi *et al* . 2019

Supplementary Table 4. Plasmids used in this study.

Supplementary Table 4. Plasmids used in this study.

Plamid ID	Name	Expressed in	Reference/Note
pKI1002	p416*GPDp-UBA1-FLAG-CYC1t*	YKI2161; *leu1* Δ	this study
pGEX-UBC4	pGEX6P-1-*GST-UBC4*	*E. coli* Rossetta 2(DE3)	Ikeuchi *et al*. 2019
pKI318	pGEX6P-2-*NOT4-FLAG*	*E. coli* Rossetta 2(DE3)	this study
pKI1026	pGEX6P-1-*His6-GST-3C-OTU2 wt*	*E. coli* Rossetta 2(DE3)	this study
pKI1027	pGEX6P-1-*His6-GST-3C-otu2-C178S*	*E. coli* Rossetta 2(DE3)	this study
pOTU-Xtal	pGEX6P-1-*His6-GST-3C-otu2-C178S (150-307)*	*E. coli* Rossetta 2(DE3)	this study
p415GPD	p415GPD (empty vector)	Y124 *otu2* Δ, Y124 *otu2* Δ*ubp3* Δ, YKI2195	lab stock
pKI1013	p415*GPDp-OTU2-3xFLAG-CYC1t*	YKI2105; *otu2* Δ, YKI2195	this study
pKI1034	p415*GPDp-otu2-C178S-3xFLAG-CYC1t*	YKI2105; *otu2* Δ, YKI2195	this study
pKI1079	p415*OTU2p-OTU2 wt-3xFLAG-CYC1t*	Y124 *otu2* Δ, Y124 *otu2* Δ*ubp3* Δ	this study
pKI1082	p415*OTU2p-otu2-C178S-3xFLAG-CYC1t*	Y124 *otu2* Δ, Y124 *otu2* Δ*ubp3* Δ	this study
pKI1151	p415*OTU2p-otu2-H300A-3xFLAG-CYC1t*	Y124 *otu2* Δ, Y124 *otu2* Δ*ubp3* Δ	this study
pKI1083	p415*OTU2p-otu2-(1-2, 71-307)-3xFLAG-CYC1t*	Y124 *otu2* Δ*ubp3* Δ	this study
pKI1117	p415*OTU2p-otu2-(1-2, 150-307)-3xFLAG-CYC1t*	Y124 *otu2* Δ*ubp3* Δ	this study
pKI1119	p415*OTU2p-otu2-(1-149)-3xFLAG-CYC1t*	Y124 *otu2* Δ*ubp3* Δ	this study
pKI1120	p415*OTU2p-otu2-(1-114)-3xFLAG-CYC1t*	Y124 *otu2* Δ*ubp3* Δ	this study
pKI1154	p415*OTU2p-otu2-K116A K117A R118A R119A-3xFLAG-CYC1t*	Y124 *otu2* Δ*ubp3* Δ	this study, BP1-mut
pKI1155	p415*OTU2p-otu2-R121A K123A R125A R129A-3xFLAG-CYC1t*	Y124 *otu2* Δ*ubp3* Δ	this study, BP2-mut

Supplementary References

1 Zivanov, J. *et al.* New tools for automated high-resolution cryo-EM structure determination in RELION-3. *Elife* **7** (2018). https://doi.org:10.7554/eLife.42166

2 Jumper, J. & Hassabis, D. Protein structure predictions to atomic accuracy with AlphaFold. *Nat Methods* **19**, 11-12 (2022). https://doi.org:10.1038/s41592-021-01362-6

3 Mevissen, T. E. *et al.* OTU deubiquitinases reveal mechanisms of linkage specificity and enable ubiquitin chain restriction analysis. *Cell* **154**, 169-184 (2013). https://doi.org:10.1016/j.cell.2013.05.046

4 Ikeuchi, K. *et al.* Collided ribosomes form a unique structural interface to induce Hel2-driven quality control pathways. *EMBO J* **38** (2019). https://doi.org:10.15252/embj.2018100276
