# Supplementary material for: Molecular basis for recognition and deubiquitination of 40S ribosomes by Otu2
Source: Nat Commun. 2023 May 12;14:2730. doi: 10.1038/s41467-023-38161-w (PMC10175282; doi:10.1038/s41467-023-38161-w)
Supplement: Supplementary file 3 — Reporting Summary [file 41467_2023_38161_MOESM3_ESM.pdf]

nature portfolio

Corresponding author(s): Prof. Roland Beckmann
Dr. Thomas Becker

Last updated by author(s): 24.03.2023

Reporting Summary

Nature Portfolio wishes to improve the reproducibility of the work that we publish. This form provides structure for consistency and transparency in reporting. For further information on Nature Portfolio policies, see our Editorial Policies and the Editorial Policy Checklist.

Statistics

For all statistical analyses, confirm that the following items are present in the figure legend, table legend, main text, or Methods section.

n/a	Confirmed	
☐	☒	The exact sample size (*n*) for each experimental group/condition, given as a discrete number and unit of measurement
☐	☒	A statement on whether measurements were taken from distinct samples or whether the same sample was measured repeatedly
☐	☒	The statistical test(s) used AND whether they are one- or two-sided<br>*Only common tests should be described solely by name; describe more complex techniques in the Methods section.*
☐	☒	A description of all covariates tested
☐	☒	A description of any assumptions or corrections, such as tests of normality and adjustment for multiple comparisons
☒	☐	A full description of the statistical parameters including central tendency (e.g. means) or other basic estimates (e.g. regression coefficient) AND variation (e.g. standard deviation) or associated estimates of uncertainty (e.g. confidence intervals)
☐	☒	For null hypothesis testing, the test statistic (e.g. *F*, *t*, *r*) with confidence intervals, effect sizes, degrees of freedom and *P* value noted<br>*Give P values as exact values whenever suitable.*
☒	☐	For Bayesian analysis, information on the choice of priors and Markov chain Monte Carlo settings
☒	☐	For hierarchical and complex designs, identification of the appropriate level for tests and full reporting of outcomes
☒	☐	Estimates of effect sizes (e.g. Cohen's *d*, Pearson's *r*), indicating how they were calculated

Our web collection on statistics for biologists contains articles on many of the points above.

Software and code

Policy information about availability of computer code

Data collection	EPU 2.12.1
Data analysis	Cryo-EM data were processed using Relion 3.0 and 3.1, MotionCor2 1.4.0, CTFFIND4 4.1.13, Gautomatch (https://www2.mrc-lmb.cam.ac.uk/?s=Gautomatch) and cryoSPARC 3.3.2. Molecular models were built and refined using COOT, version 0.8.9 and the PHENIX suite (version 1.20). Structural figures were created using ChimeraX (v.1.3.).<br>Crystal structure data were processed with XDS software (Kabsch 2010) and solved using the PHENIX suite (version 1.14). Model building was done using PHENIX and manually using COOT, version 0.8.9.<br>Acquired mass spectrometric data were analysed with MaxQuant (1.6.1.0). Data analysis for LC-MS/MS was performed with Perseus (1.5.3.2),

For manuscripts utilizing custom algorithms or software that are central to the research but not yet described in published literature, software must be made available to editors and reviewers. We strongly encourage code deposition in a community repository (e.g. GitHub). See the Nature Portfolio guidelines for submitting code & software for further information.

Data

Policy information about availability of data

All manuscripts must include a data availability statement. This statement should provide the following information, where applicable:

- Accession codes, unique identifiers, or web links for publicly available datasets
- A description of any restrictions on data availability
- For clinical datasets or third party data, please ensure that the statement adheres to our policy

The cryo-EM structural data generated in this study have been deposited in the Protein Data Bank and the Electron Microscopy Data Bank under accession codes EMD-16470 [https://www.ebi.ac.uk/emdb/search/EMD-16470] and EMD-16471 [https://www.ebi.ac.uk/emdb/search/EMD-16471] for the in vitro reconstituted Otu2-Ub-40S body and head, EMD-16525 [https://www.ebi.ac.uk/emdb/search/EMD-16525] for the Otu2-Ub-43S-PIC, EMD-16533 [https://www.ebi.ac.uk/emdb/search/EMD-16533] for the Otu2-Ub-48S-PIC, EMD-16541 [https://www.ebi.ac.uk/emdb/search/EMD-16541] for the Otu2-pre-40S, EMD-16542 [https://www.ebi.ac.uk/emdb/search/EMD-16542 for Otu2-N and EMD-16543[https://www.ebi.ac.uk/emdb/search/EMD-16543] for Otu2-C and Ub-eS7), PDB-8C83 [https://www.rcsb.org/structure/unreleased/8C83] for the Otu2-Ub-40S body, 8CAH [https://www.rcsb.org/structure/unreleased/8CAH] for the Otu2-Ub-43S-PIC, 8CAS [https://www.rcsb.org/structure/unreleased/8CAS] for the Otu2-Ub-48S-PIC and 8CBJ [https://www.rcsb.org/structure/unreleased/8CBJ] for the Otu2-pre-40S. The crystal structure of the extended OTU domain of Otu2 has been deposited in the Protein Data Bank under accession code 7PL7 [https://www.rcsb.org/structure/7PL7].

Human research participants

Policy information about studies involving human research participants and Sex and Gender in Research.

Reporting on sex and gender	N/A
Population characteristics	N/A
Recruitment	N/A
Ethics oversight	N/A

Note that full information on the approval of the study protocol must also be provided in the manuscript.

Field-specific reporting

Please select the one below that is the best fit for your research. If you are not sure, read the appropriate sections before making your selection.

☒ Life sciences ☐ Behavioural & social sciences ☐ Ecological, evolutionary & environmental sciences

For a reference copy of the document with all sections, see nature.com/documents/nr-reporting-summary-flat.pdf

Life sciences study design

All studies must disclose on these points even when the disclosure is negative.

Sample size	For mass spectrometric analyses (LC-MS/MS) followed by label-free quantification (LFQ, see Fig. 1a and 1d) three independent measurements were performed from each pullout sample (Otu2-TEV and Ubp3-TEV). After quantification and identification of hits, missing values were imputed from a normal distribution (width, 0.3; down-shift, 1.8). To test for differentially abundant proteins, a two-sided T-test was employed. Multiple testing correction was performed with a permutation-based FDR estimation (FDR<0.05). q-values≤0.05 with log2-fold changes < -0.6 and > 0.6 were considered as statistically significant.<br>For cryo-EM analysis, the sample size (number of micrographs and particles) is given in data processing workflows (Supplementary Fig. 3 and Supplementary Fig. 4f). All other experiments (debiquitination assay in Fig. 3h; sucrose density gradients followed by Western blot analysis in Fig. 4e, Supplementary Fig. 2a-2c. and Supplementary Fig. 9 , in vitro ubiquitination assays in Supplementary Fig. 4a-4e, Northern Blots analysis in Supplementary Fig. 8c were performed at least in triplcates.
Data exclusions	No data were excluded intentionally.
Replication	All experiments except for cryo-EM data analysis were performed at least three times independently and all replication attempts were successful.
Randomization	In this work, no experiments were performed where randomization is relevant.
Blinding	In this work, no experiments were performed where blinding is relevant.

Reporting for specific materials, systems and methods

We require information from authors about some types of materials, experimental systems and methods used in many studies. Here, indicate whether each material, system or method listed is relevant to your study. If you are not sure if a list item applies to your research, read the appropriate section before selecting a response.

Materials & experimental systems

n/a	Involved in the study
☐	☒ Antibodies
☒	☐ Eukaryotic cell lines
☒	☐ Palaeontology and archaeology
☒	☐ Animals and other organisms
☒	☐ Clinical data
☒	☐ Dual use research of concern

Methods

n/a	Involved in the study
☒	☐ ChIP-seq
☒	☐ Flow cytometry
☒	☐ MRI-based neuroimaging

Antibodies

Antibodies used

Anti-FLAG antibody (1:5,000; Monoclonal ANTI-FLAG M2-peroxidase, Cat# A8592, Sigma]
Anti-HA antibody (1:5,000; anti-HA-peroxidase, high Affinity, 3F10, Cat# 1201381900, Roche]
Anti-His antibody (1:1,000; anti-His tag antibody (mouse monoclonal, close HIS.H8], Cat#G020, abm]
Anti-digoxigenin antibody (1:10,000; anti-digoxigenin-AP, Fab-fragment, sheep polyclonal, Cat# 11093274910, Roche)

Validation

We used monoclonal Anti-FLAG (clone M2)-peroxidase antibody from Sigma for detection of FLAG-tagged Otu2 by Western Blotting after polyribosome profile analyses (Fig. 4e, Suppl. Fig. 2a and Suppl. Figs. 9a and 9b) and to check for presence in yeast cell lysates (Suppl. Fig. 8a).

We used Anti-HA-peroxidase antibody, clone 3F10, from Roche for detection of HA-tagged (ubiquitinated) eS7 via Western Blot in in vitro (de-) ubiquitination assays (Suppl. Fig. 4b-4d), for polyribosome profile analyses (Suppl. Fig. 2b and Suppl. Figs. 9a and 9b) and to o check for presence in yeast cell lysates (Suppl. Figs. 8a) and after affinity purifications (Suppl. Fig. 8b).

We used mouse monoclonal Anti-His antibody (clone HIS.H8) from abm to detect His-tagged Rio2 via Western Blotting after affinity purification (Suppl. Fig. 8 and 8b).

We used sheep polyclonal Anti-digoxigenin antibody from Roche to detect DIG-labeled probe hybridized to RNA on membrane via Northern Blotting to detect 20S pre-RNA and SCR1 RNA in Supplementary Fig. 8c.
